# Clinical, serological and DNA testing in Bengo Province, Angola further reveals low filarial endemicity and opportunities for disease elimination

**DOI:** 10.1016/j.parepi.2020.e00183

**Published:** 2020-09-23

**Authors:** Rossely Paulo, Miguel Brito, Pedro Van-Dunem, António Martins, Robert J. Novak, Benjamin Jacob, David M. Molyneux, Thomas R. Unnasch, J. Russell Stothard, Louise Kelly-Hope

**Affiliations:** a Centro de Investigacao em Saude de Angola(CISA)/Health Research Centre of Angola, Caxito, Angola; b Department of Tropical Disease Biology, Liverpool School of Tropical Medicine, Liverpool, UK; c Health and Technology Research Center (H&TRC), Escola Superior de Tecnologia da Saúde de Lisboa, Instituto Politécnico de Lisboa, Portugal; d National Directorate of Public Health, Ministry of Health, Angola; e College of Public Health, University of South Florida, Florida, USA

**Keywords:** Loiasis, *Chrysops*, Onchocerciasis, Lymphatic filariasis, Co-infection, Mapping

## Abstract

The prevalence of *Loa loa, Onchocerca volvulus* and *Wuchereria bancrofti* infections in an under-surveyed area of Bengo Province, Angola, was determined by surveying 22 communities with a combination of clinical, serological and DNA diagnostics. Additional information was collected on participants' duration of residency, access to mass drug administration, knowledge of insect vectors and use of bednets. A total of 1616 individuals (38.1% male: 61.9% female), with an average age of 43 years, were examined. For L. *loa,* 6.2% (*n* = 100/16616) individuals were found to have eyeworm, based on the rapid assessment procedure for loiasis (RAPLOA) surveys, and 11.5% (*n* =178/1543) based on nested PCR analyses of venous blood. *L. loa* prevalences in long-term residents (>10 years) and older individuals (>60 years) were significantly higher, and older men with eyeworm were better informed about *Chrysops* vectors. For *O. volvulus*, 4.7% (*n* = 74/1567) individuals were found to be positive by enzyme-linked immunosorbent assay (Ov 16 ELISA), with only three individuals reporting to have ever taken ivermectin. For *W. bancrofti,* no infections were found using the antigen-based immunochromatographic test (ICT) and real-time PCR analysis; however, 27 individuals presented with lymphatic filariasis (LF) related clinical conditions (lymphoedema = 11, hydrocoele = 14, both = 2). Just under half (45.5%) of the participants owned a bednet, with the majority (71.1%) sleeping under it the night before. Our approach of using combination diagnostics reveals the age-prevalence of loiasis alongside low endemicity of onchocerciasis and LF. Future research foci should be on identifying opportunities for more cost-effective ways to eliminate onchocerciasis and to develop innovative surveillance modalities for clinical LF for individual disease management and disability prevention.

## 1 Introduction

Of the main filarial nematode diseases in sub-Saharan Africa, onchocerciasis caused by *Onchocerca volvulus,* and lymphatic filariasis (LF) caused by *Wuchereria bancrofti* account for the greatest public health burden (World Health Organization, 2012a). Onchocerciasis and LF are both endemic in the Republic of Angola and the national neglected tropical disease (NTD) programme is working to scale-up preventive chemotherapy, by mass drug administration (MDA), to interrupt transmission with the long-term aim of disease elimination (World Health Organization, 2016). However, there are several challenges, especially in areas where loiasis, caused by the filarial nematode *Loa loa* exists (Vinkeles et al., 2020). In high L. *loa* prevalence communities, large-scale MDA of ivermectin is not advised as life-threatening serious adverse events (SAEs) can occur in individuals with high levels of L. *loa* microfilaraemia (Gardon et al., 1997; Zouré et al., 2011). This is of concern in low prevalence onchocerciasis areas (*i.e.* hypo-endemic), that are co-endemic with a high prevalence of loiasis areas (*i.e.* hyper-endemic), as ivermectin is not recommended and no safe, scalable alternative interventions are available (Bockarie et al., 2013).

In the northern Province of Bengo, Angola, there are areas where loiasis, onchocerciasis and LF are considered hypo-endemic, which hampers current and future preventive chemotherapy activities of the NTD programme (Zouré et al., 2011; Casaca, 1966; Brito et al., 2017). The most recent survey was conducted in 2014 using rapid clinical mapping methods, including the rapid epidemiological assessment of onchocerciasis (REMO) (Noma et al., 2002), rapid assessment procedure for loiasis (RAPLOA) (Wanji et al., 2012) and LF clinical indicators. Relatively low prevalences of loiasis (2.0%), onchocerciasis (5.3%) and LF (1.7% lymphoedema; 0.8% hydrocele) were found, with no distinct geographical distribution. Nonetheless, this survey provided some key preliminary data to the national programme, highlighting the need for further work to define community risk.

Standard epidemiological surveys traditionally use parasitological methods for detection of onchocerciasis (skin biopsies) or LF (night blood thick films) but these techniques can be impractical, being labour intensive, insensitive, time consuming and/or invasive (Wanji et al., 2016). To overcome the limitations of conventional microscopic diagnosis for filarial species identification, serological and molecular diagnosis methods have been used as alternatives with varying levels of success (World Health Organization, 2012a; Molyneux et al., 2016). In 2014, the main alternative method to serodiagnosis of loiasis was laboratory-based polymerase chain reaction (PCR), for onchocerciasis the enzyme-linked immunosorbent assay (Ov16 ELISA) and for LF the rapid diagnostic immunochromatographic test (ICT) and laboratory-based PCR (Weil and Ramzy, 2007; Kelly-Hope et al., 2018a). Of note, these alternative methods are yet to be used in conjunction with one another and none have not been used so far in Angola.

As a follow-up of the first filarial clinical mapping survey in Dande Municipality, Bengo Province (Brito et al., 2017), the objective of this current study was to examine the prevalence of filarial infections across the study areas using a combination of serological and molecular methods.

## 2 Methods

### 2.1 Study site and sampling

The study was conducted in the Centro de Investigacao em Saúde de Angola/Health Research Centre of Angola (CISA) Health Demographic Surveillance System (HDSS-Dande), area of Dande Municipality, in the north-western province of Bengo, Angola (Rosário et al., 2019) as a follow on from Brito et al., (2017). In this current study, 22 communities across the CISA area were selected on the basis of their proximity to the rivers and vegetation to help target communities that might be at higher risk of onchocerciasis and loiasis.

In each community, at least 100 individuals aged ≥15 years were approached and invited to participate in the survey by the field teams through house-to house visits. Following consent, a short questionnaire was administered in either Portuguese or Kimbundo, the official and local languages, respectively. Demographic information was collected on the individual's age, sex, length of residency and on clinical indicators for loiaisis (eye worm) and LF (limb lymphoedema, scrotal swelling due to hydrocoele). In addition, individuals were asked if they had: i) seen the vector of L. *loa*, which was shown to them in a photograph, to better understand if the *Chrysops* spp. was in the area; ii) taken MDA for onchocerciasis as some communities were in a community directed treatment for ivermectin area (CDTI) defined by APOC and; iii) a mosquito net and had used it the night before.

To determine prevalence of filarial infection through serological and molecular methods, 200 μL blood from each individual was collected by venous puncture and stored in EDTA tubes. In the field during the survey, 100 μL blood was used for LF detection using a rapid diagnostic test, and the remaining samples were kept in cooler boxes, before reaching the laboratory where two dried blood spots were prepared on Whatman 3 M filter paper and the kept at 4 °C. The remaining 100 μL blood was used for both LF real-time PCR and L. *loa* nested PCR.

### 2.2 Methods to infer prevalence

To determine the prevalence of L. *loa,* first the RAPLOA survey method was used, which is based on an individual's history of eye worm. A restricted definition was defined as individuals answering positively to a three question survey including: i) their experience;, ii) recognition of an eye worm in a photograph and; iii) a recent episode of eye worm lasting between 1 and 7 days (Zouré et al., 2011; Takougang et al., 2002).

Second, molecular biological methods were used to detect L. *loa*. Genomic DNA from the whole blood EDTA tubes using the QIAamp® DNA Blood Kit (Qiagen, Hilden, Germany). Nested PCR for L. *Loa* detection was adapted from Jimenéz et al., (2011). Briefly, a first PCR, targeted a 475 bp region of the ribosomal Internal Transcribed Space (ITS) DNA region common to different nematode species was performed and visualized in 2.0% agarose gel electrophoresis. Positive samples were used in a second PCR with L. *loa* specific primers giving 143 bp fragment, visualized in 2.0% agarose gel electrophoresis, confirming the *L Loa* infection*. L. loa* positive control was kindly provided by Professor Samuel Wanji, University of Buea, Cameroon.

To determine prevalence of the onchocerciasis parasite *O. volvulus*, dried blood spots were exported to the University of South Florida, US for analysis of *O. volvulus* antigens by the Ov16 ELISA method. Blood spots were eluted and examined for the presence of IgG4 antibodies recognizing the Ov16 antigen by ELISA, following standard procedures as described in Oguttu et al., (2014). Data on the presence of nodules using the REMO method was not used in the study, so we were unable to compare REMO survey data with the ELISA result (Brito et al., 2017). This is acknowledged as a limitation of the study.

To determine the prevalence of LF, first, the presence of main clinical conditions including limb lymphoedema (tissue swelling or thickening), and hydrocoele (scrotal swelling) was identified by the field team and verified by a local medical officer. No severity or staging of the condition was recorded. Second, the prevalence of *W. bancrofti* antigen was determined using the rapid diagnostic immunochromatographic test (ICT) card (BinaxNOW Filariasis, (Alere, Portland, ME) with 100 μL blood). Tests were conducted during the survey using the blood collected. At the time of the survey in 2014, the ICT diagnostic was the standard recommended test for determining LF endemicity (World Health Organization, 2011), however, since then several studies have shown that there is a cross-reactivity problem with the ICT diagnostic in communities with high L. *loa* prevalence, which can lead to false positives (Bakajika et al., 2014; Pion et al., 2016). To account for possible cross-reactivity in the ICTs, the prevalence of *W. bancrofti* DNA was determined using Genomic DNA extracted from the whole blood EDTA tubes using the Qiamp DNA Blood kit (Qiagen, Hilden, Germany). Real-time PCR using specific primers and a Taqman® probe was used to detect *W. bancrofti* following Rao et al., (2006), with thermal cycling conditions on a Bioarad CFX connect real time system instrument (Biorad). Amplification controls included water (no DNA template) as a negative control, and gDNA from *W. bancrofti* microfilariae as a positive control sample. *W. bancrofti* positive control was kindly provided by Professor Thomas R. Unnasch, University of South Florida, USA.

### 2.3 Data analysis and mapping

All survey data were entered into Microsoft Excel 2010 by the CISA team, and the statistical software programme IBM SPSS statistical version 21 was used for analysis. First, the prevalence for each community were quantified and mapped using ArcGIS 10.7 (ESRI, Redland CA). Second, the prevalence by length of residency (<5, 5–9, > 10 years), sex (male, female), and age group (15–19, 20–29, 30–29, 40–49, 50–59, ≥60) were summarised and statistical differences examined using the chi-square test (*p*-value considered significant at <0.05). Finally, data were examined overall and by sex and age groups (above/below mean) to assess the relationship between i) *L. loa* eyeworm and infection and the knowledge of the *Chrysops* vector ii) onchocerciasis and history of MDA and iii) LF and bed net ownership and usage.

### 2.4 Ethics and consent

The study was approved by the Angolan National Ethics Committee, administrative approval from the Ministry of Health of Angola and the Liverpool School of Tropical Medicine Research Ethics Committee (Protocol 14.022). Written informed consent was obtained from each individual and was orally explained if they were illiterate. For those who refused to participate, no further questions were asked and no information was recorded. All people identified with clinical conditions or infections were informed by the survey team or local community health worker and referred to the local health clinic for advice on self-care and treatment.

## 3 Results

**Fig. 1:**
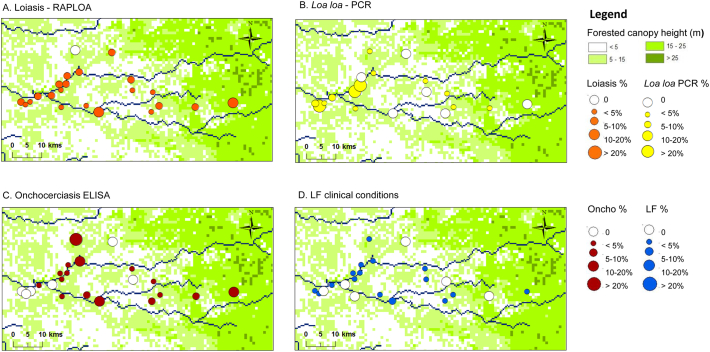
Loiasis, onchocerciasis and LF prevalence distributions in CISA communities. A. Loiasis - RAPLOA B. Loa loa - PCR C. Onchocerciasis - ELISA D. LF clinical conditions

The field survey was conducted in August 2014. In total, 22 communities, including 1616 individuals (10 to 168 per community) were surveyed across the CISA area of Dande Municipality (Table 1.). In the majority of communities, it was difficult to reach the targeted enrollment of 100 individuals, as people were either not present in the community or busy at work. Overall, there were 615 males (38.1%) and 1001 females (61.9%) included in the survey, with an average age of 43 years, ranging from 15 to 90 years. The distribution of the communities and prevalence rates are shown in Fig. 1 A–D.

**Table 1:**
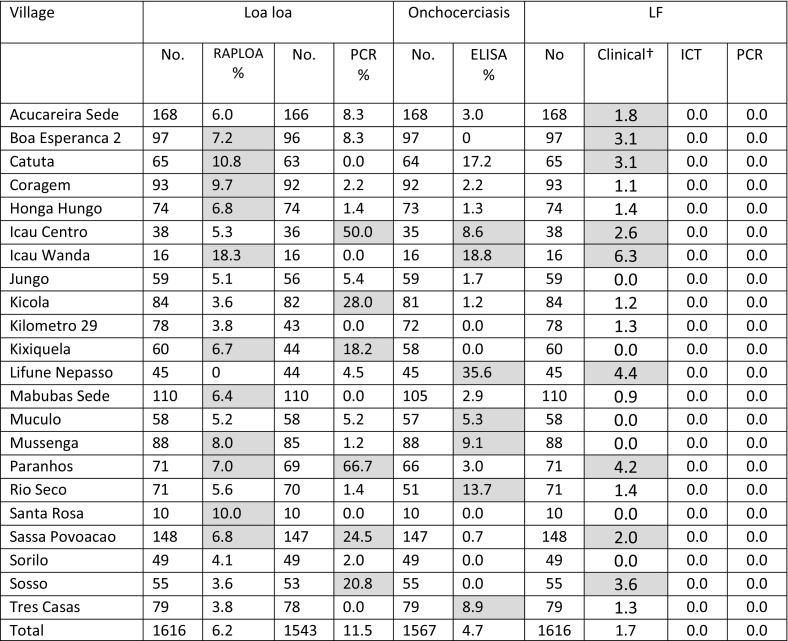
Summary of loiasis, onchocerciasis and LF serological and molecular prevalence by community.

Note: Grey shade indicates above average prevalence measures.

† Includes limb lymphoedema and/or hydrocoele (scrotal swelling).

For RAPLOA, 6.2% of 1616 individuals surveyed were found to have eye worm based on the restricted eye worm definition in 21 communities (Table 1). The highest prevalence rates were found in Icau Wanda (18.3%) and Catutua (10.8%), while none were reported from Lifune Nepasso (0.0%; Fig. 1A). Prevalence increased signficantly with length of residency from 3.0% in individuals living <5 years to 7.8% in individuals living ≥10 years in the community (Chi-square = 10.962, *p* = 0.012) (Table 2). Overall, there was no signficant difference between male 5.2% and female 6.8% prevalence rates. However, significant differences by age group overall (Chi-square = 30.1 *p* < 0.001) and among females (Chi-square = 28.66, p < 0.001) were found, with the highest prevalences in the >60 year age group (Table 3). When asked about the main *Chrysops* spp. vector, 11.2% (*n* = 179) of individuals recognized the fly when presented with a photograph. Overall, males (12.8%), individuals aged >43 years (27.3%) and those with eye worm (23.4%) were twice as likely to recognize the fly than the related subgroups (females 9.2%; aged <43 years 13.9; no eye worm 11.4%). Males with eye worm history were more likely to recognize the *Chrysop* vector in comparison with those with no eye worm history (Table 4).

**Table 2:** Summary of loiasis, onchocerciasis and LF by length of residency†.

Village	RAPLOA		L. loa		Oncho		LF	
	N	%	N	%	N	%	N	%
< 5 years	401	3.0	364	12.4	380	3.4	401	0.7
5 to 9 years	421	6.2	386	8.8	401	5.7	421	0.5
≥ 10 years	791	7.8	793	12.5	777	4.9	791	1.4
p = 0.012			Ns		Ns		Ns	
Total	1616	6.2	1543	12.0	1567	4.7	1613	0.9

† 3 missing answers.

**Table 3:** Prevalence of loiasis, onchocerciasis and LF overall and by sex and age group

		RAPLOA		L. loa PCR		Onchocerciasis		LF clinical†	
	Age class	N	%	N	%	N	%	N	%
Overall	15 to 19	190	2.6	78	21.9	176	2.3	190	1.1
	20 to 29	313	3.2	301	15.6	305	5.6	313	0.3
	30 to 39	247	4.0	235	11.5	239	5.4	247	0.0
	40 to 49	249	5.6	235	7.2	241	4.1	249	2.0
	50 to 59	307	7.8	294	7.5	300	5.0	307	1.6
	>60	310	11.9	300	8.7	306	4.9	310	1.0
		p=0.000		p=0.000		Ns		Ns	
Male	15 to 19	86	2.3	81	22.2	77	3.9	86	2.3
	20 to 29	103	3.9	100	18.0	98	4.1	103	0.0
	30 to 39	90	4.4	86	8.1	85	4.7	90	0.0
	40 to 49	96	6.3	94	9.6	91	6.6	96	4.2
	50 to 59	124	4.8	120	5.8	118	4.2	124	3.2
	>60	116	8.6	112	7.1	114	6.1	116	1.7
		Ns		p=0.001		Ns		Ns	
Female	15 to 19	104	2.9	97	21.6	99	1.0	104	0.0
	20 to 29	210	2.9	201	14.4	207	6.3	210	0.5
	30 to 39	157	3.8	149	13.4	154	5.8	157	0.0
	40 to 49	153	5.2	141	5.7	150	2.7	153	0.7
	50 to 59	183	9.8	174	8.6	182	5.5	183	0.5
	>60	194	13.9	188	9.6	192	4.2	194	0.5
		P=0.000		p=0.002		Ns		Ns	

† Includes limb lymphoedema and/or hydrocoele (scrotal swelling) in overall measure and for males.

**Table 4:** Summary of the knowledge of *Chrysops* vector overall and by sex and age group.

Village	Have you seen this fly?	RAPLOA		L. loa PCR	
Overall		N†	%	N††	%
	Yes	179	11.2	168	9.5
	No	1301	5.1	1251	12.2
		p=0.001		Ns	
					
Male	Yes	94	12.8	62	11.3
	No	466	3.6	477	17.2
		p=0.000		Ns	
					
Female	Yes	85	9.4	106	8.5
	No	835	5.9	774	9.0
		Ns		Ns	
					
<43 yrs	Yes	20	10.0	62	11.3
	No	747	9.2	477	17.2
		Ns		Ns	
					
>43 yrs	Yes	66	27.3	106	8.5
	No	647	13.9	774	9.0
		p=0.004		Ns	

† 1480 included as 136 answers missing.

†† 1419 included as 120 answers missing.

For L. *loa* nested PCR, 11.5% of 1543 individuals tested were found to be positive in 16 communities (Table 1). The highest rates were found in Icau Centro (50.0%), and Paranhos (66.7%); no positives found in Catuta, Icau Wanda, Kilômetro 29, Mabubas Sede, Santa Rosa and Três Casas (Fig. 1B). Prevalence did not signficantly differ with length of residency (Table 2) or by sex (males 11.3%; females 11.7%). However, signficant differences by age groups were found overall (Chi-square = 36.708, *p* = 0.000) and among males (chi-square = 22.051, *p* = 0.001) and females (chi-square = 19.517, *p* = 0.002), with the highest prevalences in the 15–19 year age group (Table 3). Taking infections status into account, when asked about the main *Chrysops* spp. vector, no signficant differences were found by sex and age (Table 4). Overall, the relationship between the RAPLOA and nested PCR was not significant with only eight RAPLOA positive individuals also positive by PCR.

For onchocerciasis, 4.7% of 1567 individuals tested were found to be positive in 16 communities (Table 1). The highest prevalence rates were found in Lifune Nepasso (35.6%) and Icau Wanda (18.8%), while no positves were detected in Boa Esperança 2, Kilômetro 29, Kixiquela, Santa Rosa, Sorilo and Sosso (Fig. 1C). Prevalence did not signficantly differ with length of residency or by sex (males 5.0%; females 4.6%) overall (Table 2). Similarly, no significant differences by age group overall or among males and females were found (Table 3). Three individuals (0.2%) from Coregem (female, 59 years), Hongo Hungo (male, 41 years, positive ELISA) and Lifune Nepasso (male, 21 years) reported they had taken a drug for onchocerciasis, 68.8% reported they had not, and the remaining individuals did not know.

For LF, none of the 1616 individuals surveyed were found to be positive for LF antigen or by real-time PCR (Table 1). However, 1.7% were found to have lymphoedema and/or hydrocoele in 16 communities (Table 1). In total, 11 cases of lymphoedema (0.68%; male = 6 cases, 5 females), 14 cases of hydrocoele in men (0.87%) and two men with both conditions (0.12%). The highest number of cases were identified in Sassa Povoação (3 lymphoedema; 1 hydrocoele), Açucareira Sede (1 lymphoedema, 2 hydrocoele) and Boa Esperança 2 (3 hydrocoele), Sosso (2 lymphoedema,1 hydrocoele) and Catutua (1 lymphoedema and 1 hydrocoele), Lifune Nepasso (1 lymphodema and 1 hydrocoele). One lymphodema case was found in Coragem, Icau Centro, Kilômetro 29, and Rio Reco and one hydrocoele case found in Honga Hunga, Icau Wanda, Kicola, Mabubas Sede, and Três Casas. Prevalence did not significantly differ with length of residency or for lymphoedema by sex (males 1.2%; females 0.6%) (Table 2). Similarly, no significant differences by age group overall or among males and females were found (Table 3).

Overall, 735 individuals (45.5%) reported they owned a bed net, 860 (53.2%) did not and 21 (1.2%) did not answer the question. There were significant differences between males (50.1%) and females (43.6%) and between younger (43.1%) and older (49.3%) individuals (Chi-square = 6.28, *p* = 0.012), but not between individuals affected and not affected by LF clinical condtions. With respect to bednet usage, 71.1% of those who owned a bed net reported they slept under it the previous night. Overall, there were signficant differences between males (75.2%) and females (68.2%)(Chi-square = 4.151, *p* = 0.04) in bed net usage, but not by age or clinical condition.

## 4 Discussion

Our survey used a combination of clinical, serological and molecular diagnostics to confirm the low levels of filarial endemicity across the CISA study area within Dande Municipality (Brito et al., 2017). This will help to inform the Angola NTD programme on appropriate, safe and better tailored intervention strategies locally, taking the widespread loiasis prevalence into account. This is particularly important for onchocerciasis, which was found to be hypo-endemic as previously reported (Brito et al., 2017) (see supplementary file 2). As a result ivermectin MDA is not recommended due to the risk of SAEs (Zouré et al., 2011). Currently, no alternative drugs are available for these hypo-endemic onchocerciasis ‘hotspot’ areas, and only a few individuals indicated that they had taken ivermectin, so the SAE risk is relatively unknown. However, the CISA area may be suitable for the new Test-and-Not-Treat (TNT) strategy that includes the use of the Loascope to help make a diagnosis and direct the most appropriate treatment regimen depending on the individual's infection status (Kamgno et al., 2018; Boussinesq et al., 2018). This area was previously considered to be a high-risk area for SAEs (Takougang et al., 2002). Therefore, further confirmation of the onchocerciasis distribution through skin snipping or use of other diagnostics (Kelly-Hope et al., 2018a) may be neccesary. Before or during an alternative strategy such as TNT is conducted.

The prevalence of loiasis using the RAPLOA method was found to be widespread, and higher than in the previous survey by Brito et al., (2017) (Supplementary Table 1). The reason for this difference in prevalence is unclear, but may be due to different populations being sampled, seasonality and people being away for work at different times of the year. This is a mobile population that frequently visits the town Caxito (Rosário et al., 2019). The difference between loiasis and the L. *loa* nested-PCR prevalence was also surprising with little correlation between the tests and higher L. *loa* PCR prevalence in younger age groups. The implications of this and how it relates to L. *loa* mf intensity and risk SAEs is unclear as this study was limited and did not include parasitological analysis; however it may suggest that the positive results in younger population indicate recent transmission of L. *loa* in the area. Furthermore, it may be that younger people who are infected may be new or mobile residents and not exposed enough to experience eyeworm, which was more evident in longer term and older residents. The higher risk of eyeworm in positive and older individuals, especially males, may be related to their occupation and visits to forested areas as they were more likely to recognize the *Chrysops* vector*,* also known as the readily identifiable Red Fly in Africa (Kelly-Hope et al., 2017a). More frequent forest visits have been associated with an increased risk in loiasis (Mischlinger et al., 2018; Brant et al., 2018), and using local knowledge to determine risk zones in communities and workplaces may also help to identify vector habitats. Currently, no data exists on the *Chrysops* vector in Angola (Kelly-Hope et al., 2017a), however *C. dimidiata* and *C. longicornis* have been implicated (Hawking, 1974), which may have different ecological niches to other parts of Central Africa.

The lack of LF serological and molecular positive individuals and the few related clinical cases further indicates that this a non-endemic or very low endemic area for LF, which is in accordance with historical data and recent mapping surveys from 2005 and 2010 (Casaca, 1966; Brito et al., 2017), and as such may not require MDA. It is also possible that the lymphoedema and hydrocoele cases are not related to LF, and different clinical algorithms may be needed in areas of uncertainty (Deribe et al., 2018). It is important that Angola's NTD programme uses this data to consult with the WHO as it could help to shrink the map by reducing the number of endemic municipalities throughout the country. This will save time and resources and consider more appropriate surveillance strategies for low prevalence areas (Riches et al., 2020; Kelly-Hope et al., 2017b), especially in loiasis co-endemic areas where there is increasing evidence of low LF prevalence (Wanji et al., 2019; Kelly-Hope et al., 2018b). Engagement with the national malaria control programme to help increase the bed net coverage in the area will be critical as this can reduce *W. bancrofti* transmission (Rebollo et al., 2015; Berg et al., 2012; Bockarie et al., 2009), and is a WHO recommended alternative strategy in loiasis co-endemic areas (World Health Organization, 2011; World Health Organization, 2012b). Overall, the number of patients was low and health workers need to be trained to provide care to patients, including home-based self-care for lymphoedema, with subsequent referral if needed for surgical management of hydrocele, as it can improve patient economic and quality of life outcomes (World Health Organization, 2013; World Health Organization, 2019; Betts et al., 2020).

This study highlights an integrated approach including how an alternative range of molecular and serological diagnostic tools can better define the filarial prevalence across the CISA study areas. The reason for different geographical distributions may be related to environmental factors; however, Molyneux et al., (2014) has suggested the possibility of competitive exclusion where if the three human filaria are present then their interactions might reduce the intensity of all infections due to factors such as niche separation, periodicity, immunity, geographical distribution of the parasites, ecological barriers. Nonetheless, focus now needs to be on devising opportunities to implement a practical and cost-effective strategy to eliminate onchocerciasis and conduct innovative surveillance for LF in the area. All of this will, of course, require collaboration between programmes, the community, national partners and international stakeholders (Colebunders et al., 2019).

## Declaration of Competing Interest

The authors declare that they have no competing interest.

## Appendix A Supplementary data

Supplementary material Image 1
