# Supplementary material for: Clinical, serological and DNA testing in Bengo Province, Angola further reveals low filarial endemicity and opportunities for disease elimination
Source: Parasite Epidemiol Control. 2020 Sep 23;11:e00183. doi: 10.1016/j.parepi.2020.e00183 (PMC7548300; doi:10.1016/j.parepi.2020.e00183)
Supplement: Supplementary file 1 — Supplementary material [file mmc1.docx]

**Supplementary table 1 –** Summary of loiasis distribution measured by RAPLOA methods in two surveys

| **Village** | **Survey 1** | | **Survey 2** | | | | | |
| --- | --- | --- | --- | --- | --- | --- | --- | --- |
|  | N  RAPLOA * | % Positive RAPLOA | N  RAPLOA * | % Positive RAPLOA | N  PCR | %  PCR | N Chrysops | % Chrysops |
| Açucareira Sede | 68 | 1.5 | 168 | 6.0 | 166 | 8.3 | 160 | 6.9 |
| Boa Esperança 2 | 89 | 2.2 | 97 | 7.2 | 96 | 8.3 | 93 | 15.1 |
| Bunba | 26 | 7.7 |  |  |  |  |  |  |
| Caprédio | 4 | 0.0 |  |  |  |  |  |  |
| Catuta | 31 | 0.0 | 65 | 10.8 | 63 | 0.0 | 64 | 12.5 |
| Cherú | 37 | 2.7 |  |  |  |  |  |  |
| Coragem | 67 | 1.5 | 93 | 9.7 | 92 | 2.2 | 90 | 14.4 |
| Honga Hungo | 33 | 3.0 | 74 | 6.8 | 74 | 1.4 | 71 | 18.3 |
| Icau Centro | 22 | 4.5 | 38 | 5.3 | 36 | 56.3 | 38 | 7.9 |
| Icau Wanda |  |  | 16 | 18.8 | 16 | 0.0 | 16 | 12.5 |
| Jungo | 65 | 3.1 | 59 | 5.1 | 56 | 5.4 | 49 | 22.4 |
| Kacamba | 11 | 0.0 |  |  |  |  |  |  |
| Kicola |  |  | 84 | 3.6 | 82 | 29.5 | 78 | 9.0 |
| Kilometro 29 | 20 | 10.0 | 78 | 3.8 | 43 | 0.0 | 56 | 14.3 |
| Kixiquela |  |  | 60 | 6.7 | 44 | 57.1 | 54 | 5.6 |
| Lifune Napasso | 37 | 5.4 |  |  |  |  |  |  |
| Lifune Napasso P |  |  | 45 | 0 | 44 | 4.5 | 43 | 4.7 |
| Mabubas | 95 | 1.1 | 110 | 6.4 | 110 | 0.0 | 105 | 8.6 |
| Mazaza | 18 | 0.0 |  |  |  |  |  |  |
| Muceque Teba | 18 | 0.0 |  |  |  |  |  |  |
| Muculo | 44 | 2.3 | 58 | 5.2 | 58 | 5.2 | 58 | 6.9 |
| Mussenga | 71 | 0.0 | 88 | 8.0 | 85 | 1.2 | 80 | 23.8 |
| Paranhos |  |  | 71 | 7.0 | 69 | 67.6 | 65 | 18.5 |
| Quilengues | 30 | 0.0 |  |  |  |  |  |  |
| Rio Seca |  |  | 71 | 5.6 | 70 | 1.4 | 70 | 8.6 |
| Santa Ambuleia | 41 | 0.0 |  |  |  |  |  |  |
| Santa Rosa |  |  | 10 | 1 | 10 | 0.0 | 10 |  |
| Sassa Povoação | 76 | 0.0 | 147 | 24.5 | 147 | 25.5 | 129 | 7.0 |
| Sorilo |  |  | 49 | 8.2 | 49 | 2.0 | 34 | 5.9 |
| Sosso |  |  | 55 | 0 | 53 | 20.8 | 53 | 20.8 |
| Três Casas | 74 | 1.4 | 79 | 3.8 | 78 | 0.0 | 68 | 17.6 |
| Vida e Sacrificio | 33 | 0.0 |  |  |  |  |  |  |
| **TOTAL** | **1010** | **1.8** | **1616** | **6.2** | **1543** | **11.5** | **1480** | **12.1** |

**Note:** Grey shade indicates above average prevalence measures

**Supplementary table 2 –** Summary of onchocerciasis and LF clinical conditions in two surveys

|  | Onchocerciasis | | | | LF clinical conditions | | | |
| --- | --- | --- | --- | --- | --- | --- | --- | --- |
| Village | Survey 1 | | Survey 2 | | Survey 1 | | Survey 2 | |
|  | N  REMO | % Positive REMO | N  ELISA | % Positive ELISA |  | % Positive  LF Clinical | N  LF Clinical | % Positive  LF Clinical |
| Açucareira Sede | 34 | 2.9 | 168 | 3.0 | 99 | 0.0 | 168 | 1.8 |
| Boa Esperança 2 | 66 | 7.6 | 97 | 0 | 100 | 0.0 | 97 | 3.1 |
| Bunba | 18 | 0 |  |  | 41 | 0.0 |  |  |
| Caprédio | 1 | 0 |  |  | 9 | 0.0 |  |  |
| Catuta | 21 | 9.5 | 64 | 17.2 | 36 | 0.0 | 65 | 3.1 |
| Cherú | 25 | 0 |  |  | 48 | 4.2 |  |  |
| Coragem | 19 | 0 | 92 | 2.2 | 100 | 0.0 | 93 | 1.1 |
| Honga Hungo | 7 | 42.9 | 73 | 1.3 | 46 | 2.2 | 74 | 1.4 |
| Icau Centro | 16 | 6.3 | 35 | 8.6 | 24 | 4.2 | 38 | 2.6 |
| Icau Wanda |  |  | 16 | 18.8 |  |  | 16 | 6.3 |
| Jungo | 23 | 4.3 | 59 | 1.7 | 99 | 0.0 | 59 | 0.0 |
| Kacamba | 7 | 14.3 |  |  | 25 | 8.0 |  |  |
| Kicola |  |  | 81 | 1.2 |  |  | 84 | 1.2 |
| Kilometro 29 | 10 | 10.0 | 72 | 0.0 | 27 | 7.4 | 78 | 1.3 |
| Kixiquela |  |  | 58 | 0.0 |  |  | 60 | 0.0 |
| Lifune Napasso P | 23 | 0 | 45 | 35.6 | 53 | 1.9 | 45 | 4.4 |
| Mabubas | 55 | 5.5 | 105 | 2.9 | 125 | 0.0 | 110 | 0.9 |
| Mazaza | 7 | 0 |  |  | 25 | 4.0 |  |  |
| Muceque Teba | 8 | 0 |  |  | 34 | 5.9 |  |  |
| Muculo | 18 | 0 | 57 | 5.3 | 52 | 1.9 | 58 | 0.0 |
| Mussenga | 8 | 12.5 | 88 | 9.1 | 98 | 4.1 | 88 | 0.0 |
| Paranhos |  |  | 66 | 3.0 |  |  | 71 | 4.2 |
| Quilengues | 7 | 0 |  |  | 32 | 0.0 |  |  |
| Rio Seca |  |  | 51 | 13.7 |  |  | 71 | 1.4 |
| Santa Ambuleia | 26 | 3.8 |  |  | 65 | 3.1 |  |  |
| Santa Rosa |  |  | 10 | 0.0 |  |  | 10 | 0.0 |
| Sassa Povoação | 63 | 7.9 | 147 | 0.7 | 101 | 1.0 | 148 | 2.0 |
| Sorilo |  |  | 49 | 0.0 |  |  | 49 | 0.0 |
| Sosso |  |  | 55 | 0.0 |  |  | 55 | 3.6 |
| Três Casas | 17 | 5.9 | 79 | 8.9 | 92 | 1.1 | 79 | 1.3 |
| Vida e Sacrificio | 7 | 0 |  |  | 49 | 0.0 |  |  |
| **TOTAL** | **486** | **5.1** | **1567** | **4.7** | **1380** | **1.5** | **1616** | **1.7** |

**Note:** Grey shade indicates above average prevalence measures
